# The Arithmetic of Emotion: Integration of Incidental and Integral Affect in Judgments and Decisions

**DOI:** 10.3389/fpsyg.2016.00325

**Published:** 2016-03-08

**Authors:** Daniel Västfjäll, Paul Slovic, William J. Burns, Arvid Erlandsson, Lina Koppel, Erkin Asutay, Gustav Tinghög

**Affiliations:** ^1^Linköping UniversityLinköping, Sweden; ^2^Decision ResearchEugene, OR, USA

**Keywords:** emotions, incidental affect, integral affect, judgment, decision making

## Abstract

Research has demonstrated that two types of affect have an influence on judgment and decision making: incidental affect (affect unrelated to a judgment or decision such as a mood) and integral affect (affect that is part of the perceiver’s internal representation of the option or target under consideration). So far, these two lines of research have seldom crossed so that knowledge concerning their combined effects is largely missing. To fill this gap, the present review highlights differences and similarities between integral and incidental affect. Further, common and unique mechanisms that enable these two types of affect to influence judgment and choices are identified. Finally, some basic principles for affect integration when the two sources co-occur are outlined. These mechanisms are discussed in relation to existing work that has focused on incidental or integral affect but not both.

## Introduction

Imagine walking through a park on a warm, sunny day eating your favorite ice cream. As you sit down to relax, a person from a charitable organization approaches you and asks for donations to a child in need. You are shown a photograph of 7-year-old Rokia, who is facing starvation. What information determines your decision to help Rokia? Your response is likely based in part on your current affective reaction (Schwarz, 2012). Some of the affect experienced in this situation comes from the images elicited by Rokia. Research shows that the experienced affect associated with the child in need influences judgment and decision making (Loewenstein et al., 2001; Slovic et al., 2002; Västfjäll and Slovic, 2013).

Affect stemming from a target, such as Rokia, is called integral affect or endogenous affect. Loewenstein and Lerner (2002) defined integral affect as affective influences that result from consideration of the decision or judgmental target itself. However, in many judgments other sources of affect are also present. In our donation example, unrelated or irrelevant affect (i.e., mood) elicited by the environment or the weather may also influence judgments of how we perceive the world (Schwarz, 2001). Even though the affective state is unrelated to the judgmental target, it influences judgments and decisions (Schwarz and Clore, 2004). In a famous example, Johnson and Tversky (1983) found that incidental affect (i.e., a mood state) induced by reading a newspaper article influenced subsequent risk judgments. Such influences stem from incidental or exogenous affect. Incidental affect encompasses all factors that elicit affect, but are unrelated to the judgmental target (e.g., mood, priming, motor affect, affective conditioning; Loewenstein and Lerner, 2002).

In our example, integral affect and incidental affect are both likely to influence the final judgment. However, integral affect is a “genuine” subjective reaction to a target, whereas incidental affect may be misattributed as a genuine reaction to the target or it may differentially highlight congruent affective representations of the object (Schwarz and Clore, 1983). Integral and incidental affect are often simultaneously present and jointly determine the total affective reaction to a target. We agree with Neumann et al. (2001, p. 727). claim that “…whenever a new affective stimulus is encountered, the resulting affective feeling is a function of the valence and intensity of the feeling present at the time of the encounter and the valence and intensity of the affective feeling elicited by the new stimulus”. Similarly, Bechara (2011, p. 88) writes “The effectiveness of the emotion integral to the decision-making process depends on the strength of the unrelated emotion that exists in the background”.

Thus, the donation decision may be influenced by two distinct types of affect, – one that is integral to the decision (affect elicited by considering the child and her situation) and one that is incidental to the choice (mood elicited by the sunny day). This distinction is important to consider because incidental and integral affect may have different determinants and effects on judgment. Further, confusion exists as to what the term affect means, especially in the context of influences on judgments. An illustrative example is provided by Haidt (2002) who discussed the relation between the “Affect Infusion Model” (AIM; a model of incidental affect; Forgas, 1995) and the social intuitionist model (a model of integral moral affective responses). Haidt (2002, p. 56) wrote that “…there appear to be large differences in how the AIM and the social intuitionist model explain the role of affect in moral judgment. These differences vanish, however, once it becomes clear that the two models are using the term *affect* in very different ways. If the AIM were renamed the mood infusion model, there would be no apparent contradiction”. Thus, pitting incidental against integral affect will ultimately help to clarify the affect concept.

The claim that both integral affect and incidental affect concurrently guide judgments and decisions has so far received little attention. Research on integral and incidental affect has been separate, with integral-affect research seldom considering participants’ incidental mood states (for exceptions see Peters and Slovic, 2000; Västfjäll et al., 2004) and incidental research seldom considering the integral affect that stems from the judgmental target (but see Bodenhausen et al., 2001). However, as our donation example suggests, integral and incidental affect likely co-occur on a regular basis. We suggest that incidental-integral affect interactions are common since people are always in some affective state (even a neutral mood is a piece of information; Russell, 2003). Similarly most options under consideration evoke some affect, although to different degrees (Dhar and Wertenbroch, 2000).

The goal of this paper is thus to review research on integral and incidental affect and to identify some of their common and contrasting features. From this analysis, a new set of predictions can be made that will highlight research needs and controversies in the existing literature. This analysis will also serve as a step towards a more integrative theory of the influence of different sources of affect in judgment and decision making.

### Terminology

The generic term affect is defined here as: a specific quality of “goodness” or “badness” experienced as a feeling state (with or without consciousness). This goodness–badness dimesion is often labeled “valence” (Russell, 2003). Valenced affect varies in intensity from low to high. In addition to valence, affect can be described by the arousal component of core affect (Russell, 2003) as well as the appraisals or conceptual knowledge leading up to an affective response (Barrett, 2015). This review focuses on the valence of integral and incidental affect. For a detailed discussion on the effects of arousal on judgment (see Storbeck and Clore, 2008 and for the effects of appraisal on decision making see Lerner et al., 2015).

Affective responses can occur rapidly and automatically, and may be elicited by stimulus properties, physical stimulation, perception of one’s immediate environment, thoughts and memories, or proprioceptive cues (Wyer et al., 1999). Affect informs us about our relation with the surrounding environment. From an evolutionary perspective, affect can be seen as the human alarm system. Positive affect signals that everything is safe and no specific action is needed for survival, whereas negative affect signals a potential threat and need for action. Affect thus has strong consequences for behavior and information processing. Affect has, at least, four separate roles for guiding judgment and behavior (Peters et al., 2006). First, as described above, affect can act as *information*. Second, it can act as a *spotlight* focusing us on different information. Third, affect can *motivate* us to take action or do extra work. Finally, affect may serve as a *common currency* allowing us to compare apples and oranges (Cabanac, 1992).

Mood, one form of affect, is a relatively stable and mild affective state that does not have a specific object (Frijda, 1993), whereas emotions, another form of affect, are more intense and have specific objects. In the words of Clore et al. (1994b, p. 326), “mood refers to [a] feeling state, which need not be about anything, whereas emotion refers to how one feels in combination with what that feeling is about”.

Incidental affect then is an affective state, such as a mood state, brought about by environmental or intrinsic stimulation. Integral affect, on the other hand, is elicited by perceiving the target or a mental representation of the target.

## Integral Affect

Integral affect is a pervasive aspect of daily life. We have affective feelings of different valences and intensities to most objects we encounter everyday. Integral affect is what enables us to differentiate good options from bad. Without integral affect, people would not have their favorite cookies, sports shows, or movie stars. Integral affect thus helps categorize our experiences along a good–bad dimension (Kahneman et al., 1997).

How are integral affect responses formed? Damasio (1994) argued that images become “marked” by positive and negative feelings linked directly or indirectly to somatic or bodily states. When a negative somatic marker is linked to an image of a future outcome, it sounds an alarm. When a positive marker is associated with the outcome image, it becomes a beacon of incentive. Damasio (1994) hypothesized that somatic markers increase the accuracy and efficiency of the decision process and their absence, observed in people with certain types of brain damage, degrades decision performance.

In work on the *affect heuristic* (Finucane et al., 2000; Slovic et al., 2002), we have argued that people consult or refer to an “affect pool” containing all the positive and negative tags consciously or unconsciously associated with the representation of the decision problem. Individuals consult this “affect pool” when making a judgment, rather than reviewing all available information. Slovic et al. (2002) noted that reliance on the affect heuristic is greater when deliberative capacity is limited and is less pronounced when deliberation increases. Risk judgments are often made based on this integral response (Risk-as-feelings; Loewenstein et al., 2001; Slovic and Västfjäll, 2010). Rottenstreich and Hsee (2001), for example, demonstrated that decision makers react strongly to a low probability of a strongly affective event (they value it highly) but they are insensitive to increases in that probability (its evaluation is not much higher despite its greater likelihood). The evaluation of a weakly affective event, on the other hand, grows more linearly with its probability.

Integral affective feelings are also readily accessible when making decisions. For example, Verplanken et al. (1998) demonstrated that participants responded more rapidly to their feelings than their thoughts (i.e., cognitions) about attitude objects. This accessibility is important because whatever is accessed earlier may influence later processing or directly impact behaviors (Peters et al., 2009). Ortony et al. (1988, p. 156) characterize the integral affective response (they call it “object-based emotion”) as “more immediate, more spontaneous, and less affected by accessible cognitive processes than almost all of the other emotions.”

Pham et al. (2001) suggested that integral affective responses can be elicited by three types of mechanisms: Type-I affect is based on the triggering of very basic, innate, sensory-motor programs that are important for bio-regulation (i.e., avoidance of bitter-tasting food). Type-II affect is triggered by the mapping of stimulus features onto acquired schematic structures that have been previously associated, through conditioning or episodic memory, with particular affective responses, similar to Damasio’s (1994) somatic markers. Type-III affect is based on a controlled appraisal of the stimulus that involves a subjective assessment of the stimulus’ significance for well-being.

Bodenhausen et al. (2001) further distinguished between chronic integral affect, referring to enduring affective reactions to a specific target, and episodic integral affect that contains affective reactions activated in a particular setting.

## Incidental Affect

### Incidental Moods

Incidental mood states may also be used as a heuristic for making evaluative judgments (Schwarz, 2012). The mood-as-information view assumes that, when people make evaluative judgments, they do not consult all available information but simplify judgments by using their affective reaction to the object as a basis. People ask themselves “How do I feel about it?” and, while doing this, monitor their own feelings. Current mood may be misattributed as a reaction to the target. As a result, evaluative mood-congruent judgments occur (Schwarz and Clore, 2007)^1^. For example, minor mood-influencing events such as finding a coin in a copying machine or learning that a favorite soccer team won a game influenced ratings of global subjective well-being (Schwarz, 2012).

#### Salience of Mood

Mood is a relatively salient affective state. Research on cue salience has shown that salient cues are the primary determinant of judgments (Kahneman, 2003). Schwarz (2001) therefore suggested that the more the salient mood is compared to other information relevant for the judgment, the greater the effect it will have on the judgment. Other cues may even be disregarded in the presence of a salient mood. Siemer and Reisenzein (1998) noted that mood salience is often related to increases in mood intensity.

#### Misattribution and Judgmental Correction: the Affective Gatekeeper

A central premise for affect misattribution to occur is that the affective system cannot distinguish “true” (integral) feelings from “false” (incidental) feelings, and thus treats any currently experienced affect as a reaction to the target currently attended. While much research has shown that misattribution occurs frequently in everyday life (e.g., Schwarz, 2012) there are certain conditions where misattribution may not occur – where the “affective gatekeeper” is alerted that affective information should be discounted and not treated as a genuine reaction to the target.

For instance, people only use their current feelings as a basis of judgment when it is perceived to contain valuable or diagnostic information (Pham, 2007) or when there is no cause to distrust the feeling as being a genuine reaction to the target (Schwarz, 2012). In a classical demonstration of how subtle reminders can alert the affective gatekeeper, Schwarz and Clore (1983) showed that when participants were made aware that their mood was caused by something unrelated to the judgmental target (e.g., a rainy day or misattribution manipulation), the mood-congruent effect disappeared or was discounted (Schwarz, 2012). Thus, certain cues can alert the affective gatekeeper and help to determine what information is integrated, and what information is excluded, from overall affective judgments.

Other research also suggests that this type of affective gatekeeping is the second stage in a two-stage process where available information (i.e., affect) is used as a first proxy for evaluation, then a correction or “reality check” is applied if, for some reason, the correctness of the initial evaluation is called into question (Gilbert, 2002). The effect of mood may not only be eliminated by this, but even reversed. For instance, Wegener et al. (1995) demonstrated that people tend to overcorrect when they believe that how they feel will influence their judgments. This overcorrection can lead to mood-incongruent judgments. Similarly, Pham (1998) showed that, when participants believed their feelings were of no importance for the decision, mood effects diminished. Thus, mood needs to be representative or relevant for the judgment task to enter into the judgmental process.

Siemer and Reisenzein (1998) further noted, however, that mood salience may be related to mood source awareness, in that if intensity is high (as in the case of emotions) people also often become aware of what is causing the effect. When participants become aware of the mood-producing source, incidental mood is likely to have a smaller or no effect on the subsequent judgment as the mood is viewed as irrelevant and is discounted or corrected (Schwarz, 2001).

#### Source Salience Limits the Effects of Incidental Affect on Judgments But Not the Experience

Being aware of the source of the mood may limit its influence on judgments, but does not appear to alter physiological indices of experienced affect (as measured by facial EMG) (Neumann et al., 2001). Studies of individual differences in the use of incidental affect also suggests that making the true source of the incidental affect salient does not always lead to discounting or correction. Gasper and Clore (2000), for example, demonstrated that both chronically anxious individuals and those low in attention-paid-to-feelings relied on current mood even when the true source of their affect was made salient. For those low in attention-paid-to-feelings, the misattribution manipulation served to make the mood salient and of use in judgments. This finding is in agreement with Siemer and Reisenzein’s (1998, p. 800) threshold hypothesis in which they assume that “mood has an effect on evaluative judgments only if it exceeds a certain minimum value, or threshold, of salience”. However, this conclusion is contested by a large literature showing that subliminally induced affect (“unconscious emotion”) has strong effects on evaluative judgments (Zajonc, 1980; Winkielman and Berridge, 2004).

### Incidental Motor Influences and Somatic Processes

Incidental affect includes, not only incidental moods, but also affect elicited by facial, postural, and behavior expression that may be misattributed as relevant reactions to targets (Schwarz and Clore, 2004). According to Neumann and Strack (2000), these motor and somatic processes can be divided into two groups on the basis of their experiential quality (i.e., the extent to which they elicit affect that is experienced).

#### Incidental Motor Influences

Neumann and Strack (2000) suggested that some motor tasks influence the experience of affect and that this experiential affect then mediates any effects on subsequent judgments (see also Strack, 1992). Their primary example is facially induced affect. Facially induced affect can be manipulated by having participants contract the zygomatic muscle in the cheek (Strack et al., 1988). Facilitating smiling (by having participants hold a pen between the teeth) increased the rated funniness of a cartoon, whereas inhibiting smiling (by having participants hold a pen between their lips) decreased the rated funniness of the same cartoon. Zajonc et al. (1989) showed that pronouncing vowels that facilitated smiling (the vowel e) versus vowels that inhibited smiling (the German vowel ü) produced similar results. Motor influences are not always mediated by experienced affect, however, (Neumann and Strack, 2000). For instance, isometric flexion and extension of the arm, head movements, pushing a lever toward or away from the body, and the visual impression of moving away or toward the computer screen are all tasks that influence evaluation of targets without any evidence of changes in experienced affect (Winkielman and Berridge, 2004).

### Incidental Emotions

Incidental emotions invoked by a specific eliciting event are associated with specific appraisal patterns (Lerner et al., 2015). Lerner and Keltner (2000) demonstrated that judgments of risk were differentially influenced by incidental fear and anger. Further, Lerner et al. (2004) demonstrated that sadness and disgust differentially influenced the endowment effect. Sad individuals showed a stronger effect of ownership compared to those in a neutral mood, whereas disgusted individuals demonstrated a reverse endowment effect. This research suggests that cognitive appraisals of incidental emotions may dominate behavioral responses over their experiential quality.

DeSteno et al. (2000) also demonstrated that sadness and anger, two distinct negative emotions, differentially biased likelihood estimates of sad and angering events. In addition, a reversal of the bias occurred for individuals who expended greater cognitive effort and thus may have been more aware of the source of the emotion. Similarly, Keltner et al. (1993) showed that asking participants to label their current affect as specific emotions forced them to think about the cause of the feeling, and rendered the affect uninformative for subsequent judgments of well-being.

Consequently, incidental emotions are less likely to influence judgments than incidental mood, since emotions are strong in intensity and people are highly aware of their cause. Based on research demonstrating that reminders of the cause of experienced affect reduce mood’s influence on judgments, intense incidental emotions should not influence normatively unrelated target judgments (Schwarz, 2001). However, appraisal tendencies could still have a separate effect on judgments and behaviors. In fact, the appraisal effect of incidental emotions may be resilient to misattribution manipulations (Han et al., 2010). The intensity and perceived salience of incidental emotions dissipates over time, however, leaving the individual in a diffuse mood state. After a sufficient decay of emotion intensity and a decreased focus on the cause of the affect, incidental emotions then may color unrelated judgments. Such diffuse mood states, however, may be based more on valenced affect, rather than a discrete emotion (Peters et al., 2004).

### Experiential Salience and Affect Awareness

Awareness of the affect-eliciting source may be characterized along a continuum from no awareness (e.g., approach/avoidance tasks; facially induced affect) to medium awareness (moods; often the source is not apparent, but experimental manipulations may be used to direct attention to the source) to high awareness (emotions; source is often evident.) This distinction is similar to Wyer et al.’s (1999) proposal that affect elicited by low intensity cues (e.g., proprioceptive cues) does not receive conscious attention. Other affective states, instantiated by events or appraisals, are of higher intensity and therefore noticed. Experiential salience concerns the accessibility of affect upon introspection. The experiential salience of the affect is different from awareness of the source although the two concepts sometimes are related (Siemer and Reisenzein, 1998). For instance, incidental emotions are highly salient in people’s minds, and people are quite aware of the affect-inducing source. For moods, however, experiential salience may be high while awareness of the source remains low. For experiential motor influences such as facial feedback, affect is salient (as evidenced by changes in self-reported mood; Strack et al., 1988), but source awareness is very low. Non-experiential affective tasks such as the arm flexion manipulation are low in both experiential salience and source awareness. Affect that is experientially more salient will influence judgments more. We suggest that experiential salience and source awareness are two main experiential factors that influence the use of incidental affect as information.

## The Integration of Integral and Incidental Affect

### Affect Integration: Averaging and Summation

How do incidental and integral affect combine into a single affective reaction? Ample research in impression formation shows that adding information that is moderately positive to information that is highly positive leads to lower judgments (Anderson, 1981), resulting from averaging values rather than adding them (see also Seta et al., 2008). An example of this from the consumer domain is provided by Yadav (1994), who asked consumers to rate their preference for different sets of furniture. Participants in the individual-item condition read information about a bed that pretest participants had rated as excellent. Those in the bundle condition rated a set consisting of two items: the same highly favorable bed plus a chest that was described as moderately favorable. Participants gave higher preference ratings to the bed alone than those in a separate group gave to a set containing both the bed and the moderately favorable chest (see also Weaver et al., 2012). Interestingly, Kralik et al. (2012) found evidence for similar averaging in non-human primates; rhesus monkeys preferred a high-value food item alone to the same item paired with one of positive but lower value. Similarly, Hsee et al. (1999) asked respondents to state the amount they were willing to pay to purchase each of two sets of dinnerware. Set S contained 24 pieces, all in good condition. Set J contained all of the same pieces plus 8 more, all in good condition, along with 16 other pieces that were broken (40 total). In single (separate) evaluation, respondents were willing to pay more for set S, though it was the inferior option, apparently devalued by the broken pieces. But in joint (side-by-side) evaluation, respondents were willing to pay more for Set J. Thus, in separate evaluation, negative affect appears to reduce positive affect through averaging.

But sometimes the integration goes awry in a peculiar form of affective calculus that Polish poet Herbert (2007, p. 286) has called “the arithmetic of compassion”. For example when only one life is at stake, the value attached to saving or prolonging that life is extreme. But as the number of lives at risk increases, phenomena such as psychophysical numbing and psychic numbing (Slovic, 2007), appear to lead our fast, intuitive, gut reactions on a path much different from one guided by the normal logic of arithmetic. With numbing, one life plus one life may be valued at something less than two lives. With compassion fade or collapse, 1 + 1 may be valued as less than 1 (Västfjäll et al., 2014).

In additional experiments we have studied the arithmetic of compassion in the context of what we term “pseudoinefficacy” (Västfjäll et al., 2015; see also Slovic and Slovic, 2015; Wiss et al., 2015). We have documented that positive feelings about children one can help are dampened by negative feelings associated with children who cannot be helped. Specifically, we found that pictures of the children not helped induced negative affect that reduced the positive warm glow for the child that could be helped. The stronger the negative feelings associated with those not helped, the lower the warm glow anticipated from helping a child who could be helped. We further found that the pseudoinefficacy effect is not merely due to visual distraction resulting from images of children not helped. Warm-glow ratings of a single child who could be helped were not reduced when that child was accompanied by non-affective visual distractors (e.g., a shape). In further support of an affect-based explanation, we found that when other, unrelated, pictures that induced negative emotion (taken from the International Affective Picture System; Bradley and Lang, 2000) accompanied the single child, warm-glow ratings were as low as in the pseudoinefficacy conditions where children not being helped were present. The stronger the rated negative affect toward the unrelated pictures, the lower the warm glow that was associated with helping.

Taken together, ample research suggests that different sources of affect are averaged or summed (Anderson, 1981). However, other research has found that not all forms of affect integration follow simple arithmetic laws such as averaging (Olsen and Pracejus, 2004; Chowdhury et al., 2008). To guide our discussion of how incidental and integral affect may be combined in ways that differ from averaging, we first discuss relevant research on the effects of incidental affect on judgments of integral affect. Such judgments appear to be based on the integration of affective reactions to a target (integral affect) with more free-floating incidental affect.

### Mood Effects on Judgments

In this section we examine studies that investigated the influence of incidental mood on affective judgments (i.e., well-being, liking judgments, emotion ratings) of a specific target other than the person’s mood state.

#### Congruence and Incongruence

**FIGURE 1:**
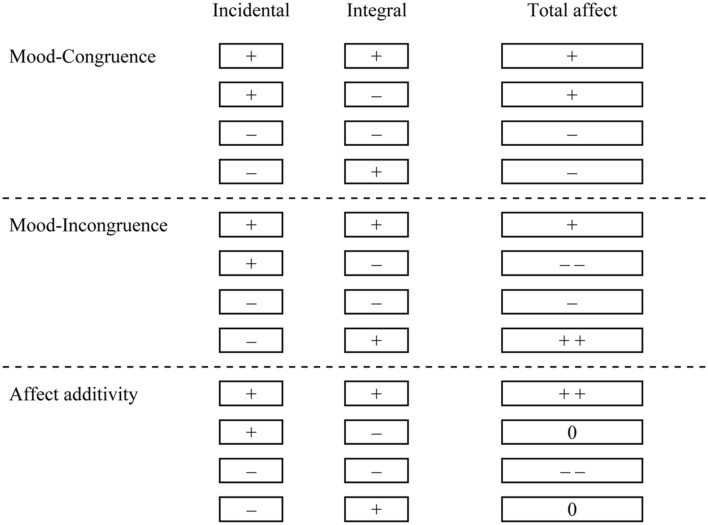
**Three different forms of affect integration previously proposed in the literature.** (1) Mood-congruent effects are obtained when the valence of the incidental affect influences the target so that positive incidental affect makes the evaluation of the target more positive, and negative incidental affect influences evaluations so that the target is perceived as more negative **(upper)**. (2) Mood-incongruent effects are obtained when positive incidental affect makes the judgmental target be perceived as more negative, than a negative incidental affect state does **(middle)**. (3) Affect additivity is the case where congruent valences of incidental and integral affect are added and incongruent valences cancel each other **(bottom)**.

Schwarz and Clore (1983) showed in an early study that current mood influenced ratings of overall well-being in a mood-congruent manner (assimilation). A later study by Siemer and Reisenzein (1998) replicated these findings using a large number of satisfaction and emotion items. Neumann et al. (2001) found that a pre-existing mood state intensified congruent emotional responses to a humorous cartoon and attenuated incongruent emotional reactions. Other research, however, suggests that current mood is sometimes contrasted with the affective valence of the judgmental target, resulting in mood-incongruent judgments (Martin, 2000). For example, Manstead et al. (1983) found that humorous movie clips were evaluated more positively if they followed a negative-affect-inducing movie than if they followed a positive-affect-inducing movie. Schwarz et al. (1987) induced pleasant or unpleasant moods by letting participants stay in a pleasant or unpleasant room. Following the mood induction, participants rated their global well-being and their satisfaction with their housing conditions (e.g., in their dormitory). A mood-congruent effect was obtained on global well-being, a mood-incongruent effect (contrast) on ratings of housing satisfaction. Participants thus used their mood as information for an overall affect judgment, but used their pleasant or unpleasant experience in the experiment as a standard of comparison or reference point to construct the specific judgment about housing. Thus, mood-incongruent effects can be obtained when a salient and meaningful standard of reference is readily available. **Figure 1** is a schematic representation of how congruency and incongruency effects may influence overall affect.

In **Figure 1**, we assume that both incidental and integral affects contribute to the overall affective reaction. The relative weight placed on incidental and integral affect, respectively, is unknown and likely to vary with situational demands and individual characteristics. Their accessibility and knowledge of their source should also influence their weights. We do, however, assign integral affect a relatively larger role in determining the total affective reaction since an integral affective reaction, by definition, is a relevant source of affect.

Mood-congruent effects are obtained when the valence of the incidental affect influences the target so that positive incidental affect makes the evaluation of the target more positive, and negative incidental affect influences evaluations so that the target is perceived as more negative. The processes underlying this effect may include a highlighting of valence-congruent attributes so that they draw more attention, are more accessible while considering the target, and thus receive more weight in the judgment (Kahneman, 2003).

Mood-incongruent effects are obtained when positive incidental affect makes the judgmental target be perceived as more negative, than a negative incidental affect state does (depicted in the middle part of **Figure 1**). Mood-incongruent judgments typically occur through motivated affect regulation, when people overcorrect for a mood-inducing bias (Schwarz, 2001) or when a salient comparison is available. Possible processes underlying this effect include increased deliberative processing due to processing focus or motivated processing to maintain or attain a positive mood or perceived well being.

### Additivity-of-Affect

Research obtaining mood-congruent or mood-incongruent effects typically has studied the effect of incidental mood, a relatively salient experiential state. Other research suggests that incidental affect that is less experientially salient may influence integral affective reactions to targets differently (Murphy et al., 1995). For instance, Neumann et al. (2001, p. 726) argued that “pre-existing moods fuse with feeling stemming from an emotion eliciting event”. Neumann et al. (2001) suggest that congruent valences should add together (see bottom row of **Figure 1**), whereas incongruent valences should cancel (“additivity-of-affect hypothesis”; note that for incongruent valences, the mood-congruence view would suggest that incidental affect would either dampen or intensify the integral affect, but not cancel). Moreover, Neumann et al. (2001) argue that only affect that is experientially salient and low in source awareness (e.g., facially induced affect, unobtrusively induced moods) should exhibit this additivity of affect. Neumann et al. (2001) investigated the influence of incidental affect (induced by having participants listen to a philosophical talk spoken either with a happy or sad voice) on integral affect (humor responses) and found that it followed the additivity-of-affect pattern: The incidental mood induction resulted in the expected mood ratings (happy and sad), but participants were not aware that the tone of voice had influenced feelings. This finding suggests that indeed the incidental affect manipulation was experientially salient and low in source awareness.

In summary, incidental affect may either influence judgments of integral affect through mechanisms of mood congruence/assimilation and mood in-congruence/contrast or according to the affect-additivity principle. We propose that a major determinant for the type of affect integration is the experiential salience and awareness of the source of incidental affect. For incidental affect that is low in source awareness [unobtrusively induced moods as in Neumann et al.’s (2001) study or facially induced affect], affect integration should follow the affect-additivity principle, whereas incidental affect higher in source awareness should follow the principles of mood congruence/incongruence.

#### An Example of Affect Integration: Integral Affect’s Role in Determining the Intensity of the Overall Response

It is possible to characterize both incidental and integral affect in terms of their experiential salience and source awareness. Whereas incidental affect can vary in both salience and awareness, we assume that integral affect is generally high in source awareness (because the judgment is about the source), but that it can vary in intensity and precision.

**FIGURE 2:**
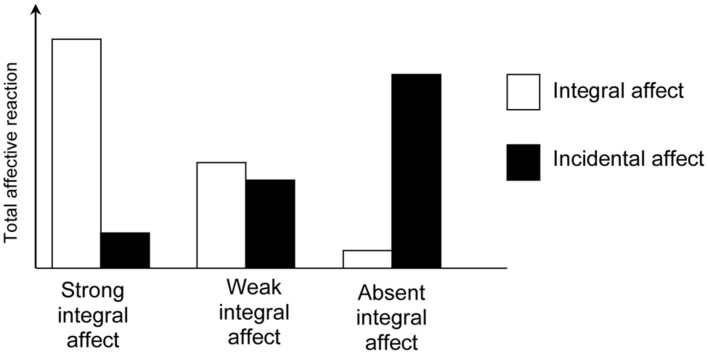
**The influence of incidental affect is contingent on the intensity of integral affect.** Left bars for strong integral affect, incidental affect has little possibility to influence the overall affective reaction. Middle bars for weak integral affect, incidental affect is allowed a relatively large influence. Right bars if integral affect is absent, incidental affect will have a stronger contribution to the overall affective reaction.

Affect intensity is the strength of the integral response to a stimulus (Frijda, 1993). Higher affect intensity will be experientially more salient. In addition, some events, objects, or thoughts elicit precise responses, in that they exhibit little variation across situations and/or individuals, whereas other responses seem to be more imprecise with larger variation. Precise integral responses are often based on vivid mental or actual images and are easily mapped onto a good–bad scale (Slovic et al., 2002). Imprecise integral responses are experienced when affect is ambiguous, due to the existence of conflicting or mixed affect (Larsen et al., 2001) or a lack of vivid images or comparison from which to draw meaning. Both the intensity and precision of the integral response are likely to determine the role of incidental affect in the overall judgment. More specifically, we argue that incidental affect will have a larger influence on judgments when the integral response is less intense and/or less precise. When integral affect is strong, incidental affect has little possibility to influence the overall affective reaction. However, when integral affect is weak, incidental affect will be allowed a larger influence. If the affect (as an experienced affective reaction per se) is absent, current incidental affect will have a stronger influence (**Figure 2**).

We found initial support for this hypothesis in a study of charitable giving (Västfjäll et al., 2008). Participants were first induced to negative or positive moods (incidental affect) and were then asked to donate money either to a target with precise/intense integral affect (a single, named individual shown in a photograph) or to a target eliciting less precise/intense integral affect (a single, unidentified statistical victim). Consistent with the hypothesis outlined above, positive-mood participants gave more than negative-mood participants only when the target elicited less precise/intense integral affect.

## Discussion and Conclusion

Two types of affect, integral and incidental, both have substantial influence on judgment and decision making. However, until now few attempts have been made to compare incidental and integral affect, but several calls for such integrative work have been issued. For instance, Perrott and Bodenhausen (2002) asked to what extent models of incidental affect can accommodate integral affect? Perrott and Bodenhausen (2002, p. 85–85) further argued that “It remains very much an open question whether these types of [integral] affect operate on the same general principles as those that have been documented in studies investigating incidental affect”. Ultimately, a full response to this question requires more empirical data. Based on existing research, the present review delineated a select number of mechanisms concerning how and when incidental and integral affect jointly influence judgments and decisions. It is our hope that this review will stimulate further empirical studies.

So far, research focusing only on incidental affect has tended either to assume that the target under consideration elicits no affect (or is affectively neutral) or it has been vague in specifying the role of integral affect. Forgas (2002) even argued that some incidental affect research explicitly predicts that integral affect should not influence judgments. It is, however, evident that integral affect is present in many studies focusing on incidental affect. The question becomes: to what extent does integral affect modify the misattribution of incidental affect? The present review suggests that both incidental and integral affect theories need to take into account both forms of affect. First, if integral and incidental are simultaneously present, we assume that integral affect will dominate the overall response. Second, our analysis suggests that it is mainly when integral affect is moderate or low in intensity that current incidental mood will have a substantial effect on the overall judgment. Third, our review suggests that incidental affect will have a substantial effect on the integral response when incidental affect is salient (but the source is not salient).

Similarly, the current review suggests that research focusing on integral affect must consider incidental affect. Several reasons for this can be highlighted. First, people are always in a more or less valenced mood state (Russell, 2003) so that incidental affect has the potential to influence most judgments. Second, it is likely that many integral responses are of moderate intensity. The functional value of integral responses would otherwise be very limited. Research on emotion intensity suggests that people seldom experience very strong affect (both incidental and integral emotions), presumably because prolonged, high-intensity experiences would ultimately exhaust the human biological system (Clore et al., 1994a). Incidental affect is likely to have its largest impact on moderate and low intensity integral responses. Third, both incidental and integral affect are both most likely to influence judgments when other judgment criteria and/or information are unavailable, and when deliberative processing is impaired (Pham, 2009). It may be argued that in such conditions, integral affect research may very well be measuring the effect of incidental affect on judgment and choice.

Given that both incidental and integral affect often are present and influence the decisions people make, what is the effect in everyday life? Our analysis suggests that integral affect often is a good proxy for preferences, and may even be a prerequisite for deriving meaning from abstract and complex decisions (Peters et al., 2009). Incidental affect, however, is of another status. Normatively, incidental affect is unrelated to the decision at hand and may therefore be considered a bias or unwanted influence (Vohs et al., 2007). But, our review suggests that incidental affect may be both beneficial and detrimental to efficient decision making (see also Pham, 2007). If incidental affect is congruent with the target, it may amplify integral affect or the overall affective reaction. If it is incongruent, it may attenuate the response. In cases where affect is considered a relevant criterion, the interaction between congruent incidental and integral affect is likely to be beneficial. When affect is less relevant or used as a heuristic due to restricted processing, incidental affect may be less beneficial. The impact of integral and incidental affect on the quality of the judgment and decisions will ultimately depend on the specific decision context. In situations where incidental affect may have a detrimental effect, the affective gatekeeper must be extra alert. An important task for future research is to more precisely identify the underlying mechanism for incidental-integral affect integration, so that debiasing procedures can be developed to mitigate possible detrimental effects.

## Author Contributions

Reviewed research and wrote the paper: DV, PS, WJB, AE, LK, EA, GT.

## Conflict of Interest Statement

The authors declare that the research was conducted in the absence of any commercial or financial relationships that could be construed as a potential conflict of interest.

## Footnotes

1 We leave aside discussion of how incidental affect influence cognitive processes (Isen, 2000) or how it motivates behavior as it is not a primary focus of the present article.

## References

[B1] Anderson N. H. (1981). *Foundation of Information Integration Theory.* New York, NY: Academic Press.

[B2] Barrett L. F. (2015). “Construction as an integrative framework for the science of emotion,” in *The Psychological Construction of Emotion*, eds Barrett L. F. Russell J. A. (New York, NY: Guilford), 448–458.

[B3] Bechara A. (2011). “Human emotions in decision making: are they useful or disruptive?,” in *Neuroscience of Decision Making*, eds Vartanian O. Mandel D. (New York, NY: Psychology Press), 73–90.

[B4] Bodenhausen G. V. Mussweiler T. Gabriel S. Moreno K. N. (2001). “Affective influences on stereotyping and intergroup relations,” in *Handbook of Affect and Social Cognition*, ed. Forgas J. P. (Mahwah, NJ: Erlbaum), 319–343.

[B5] Bradley M. M. Lang P. J. (2000). “Measuring emotion: behavior, feeling and physiology,” in *Cognitive Neuroscience of Emotion*, eds Lane R. Nadel L. (New York: Oxford University Press), 242–276.

[B6] Cabanac M. (1992). Pleasure: the common currency. *J. Theor. Biol.* 155 173–200. 10.1016/S0022-5193(05)80594-6 12240693

[B7] Clore G. L. Ellsworth P. C. Frijda N. H. Izard C. E. Lazarus R. LeDoux J. E. (1994a). “What are the minimal cognitive prerequisites for emotion?,” in *The Nature of Emotion: Fundamental Questions*, eds Ekman P. Davidson R. J. (New York, NY: Oxford University Press), 179–234.

[B8] Clore G. L. Schwarz N. Conway M. (1994b). “Affective causes and consequences of social information processing,” in *Handbook of Social Cognition*, 2nd Edn, eds Wyers R. S. Srull T. K. (Hillsdale, NJ: Lawrence Erlbaum Associates), 323–417.

[B9] Chowdhury R. M. M. I. Olsen G. D. Pracejus J. W. (2008). Affective responses to images in print advertising: affect integration in a simultaneous presentation context. *J. Advert.* 37 7–18. 10.2753/JOA0091-3367370301

[B10] Damasio A. R. (1994). *Descartes’ Error: Emotion, Reason, and the Human Brain*. New York, NY: Avon.

[B11] DeSteno D. Petty R. E. Rucker D. D. Wegener D. T. (2000). Beyond valence in the perception of likelihood: the role of emotion specificity. *J. Pers. Soc. Psychol.* 78 397–419. 10.1037/h0087886 10743870

[B12] Dhar R. Wertenbroch K. (2000). Consumer choice between hedonic and utilitarian goods. *J. Market. Res.* 37 60–71. 10.1509/jmkr.37.1.60.18718

[B13] Finucane M. L. Alhakami A. Slovic P. Johnson S. M. (2000). The affect heuristic in judgments of risks and benefits. *J. Behav. Decision Mak.* 13 1–17. 10.1002/(SICI)1099-0771(200001/03)13:1<1::AID-BDM333>3.0.CO;2-S

[B14] Forgas J. P. (1995). Mood and judgment: the affect infusion model (AIM). *Psychol. Bull.* 117 39–66. 10.1037/0033-2909.117.1.39 7870863

[B15] Forgas J. P. (2002). Towards understanding the role of affect in social thinking and behavior. *Psychol. Inq.* 13 90–102. 10.1207/S15327965PLI1301_03

[B16] Frijda N. H. (1993). “Moods, emotion episodes, and emotions,” in *Handbook of Emotion*, eds Lewis M. Haiviland J. M. (New York, NY: Guilford Press), 381–404.

[B17] Gasper K. Clore G. L. (2000). Do you have to pay attention to your feelings in order to be influenced by them? *Pers. Soc. Psychol. Bull.* 26 698–711. 10.1177/0146167200268005

[B18] Gilbert D. T. (2002). “Inferential correction,” in *Heuristics and Biases: The Psychology of Intuitive Judgment*, eds Gilovich T. Griffin D. Kahneman D. (Cambridge: Cambridge University Press), 167–184.

[B19] Haidt J. (2002). Dialogue between my head and my heart: affective influences on moral judgment. *Psychol. Inq.* 13 54–56.

[B20] Han S. Lerner J. S. Zeckhauser R. (2010). *Disgust Promotes Disposal: Souring the Status Quo*. HKS Faculty Research Working Paper Series, RWP10-021, Boston: John F. Kennedy School of Government, Harvard University.

[B21] Herbert Z. (2007). “Mr. Cogito reads the newspaper,” in *The Collected Poems 1956–1998*, ed. Valles A. (New York, NY: HarperCollins), 285–286.

[B22] Hsee C. K. Loewenstein G. Blount S. Bazerman M. H. (1999). Preference reversals between joint and separate evaluations of options: a review and theoretical analysis. *Psychol. Bull.* 125 576–590. 10.1037/0033-2909.125.5.576

[B23] Isen A. M. (2000). “Positive affect and decision making,” in *Handbook of Emotions*, 2nd Edn, eds Lewis M. Havieland J. M. (London: Guilford), 417–435.

[B24] Johnson E. J. Tversky A. (1983). Affect, generalization, and the perception of risk. *J. Pers. Soc. Psychol.* 45 20–31. 10.1037/0022-3514.45.1.20

[B25] Kahneman D. (2003). A perspective on judgment and choice: mapping bounded rationality. *Am. Psychol.* 58 697–720. 10.1037/0003-066X.58.9.723 14584987

[B26] Kahneman D. Wakker P. Sarin R. (1997). Back to bentham? Explorations of experienced utility. *Q. J. Econ.* 112 375–406. 10.1162/003355397555235

[B27] Keltner D. Ellsworth P. Edwards K. (1993). Beyond simple pessimism: effects of sadness and anger on social perception. *J. Pers. Soc. Psychol.* 64 740–752. 10.1037/0022-3514.64.5.740 8505705

[B28] Kralik J. D. Xu E. R. Knight E. J. Khan S. A. Levine W. J. (2012). When less is more: evolutionary origins of the affect heuristic. *PLoS ONE* 7:e46240 10.1371/journal.pone.0046240 PMC3463577 23056270

[B29] Larsen J. T. McGraw A. P. Cacioppo J. T. (2001). Can people feel happy and sad at the same time? *J. Pers. Soc. Psychol.* 81 684–696. 10.1037/0022-3514.81.4.684 11642354

[B30] Lerner J. S. Keltner D. (2000). Beyond valence: toward a model of emotion-specific influences on judgment and choice. *Cogn. Emot.* 14 473–493. 10.1080/026999300402763

[B31] Lerner J. S. Li Y. Valdesolo P. Kassam K. (2015). Emotion and decision making. *Annu. Rev. Psychol.* 66 799–823. 10.1146/annurev-psych-010213-115043 25251484

[B32] Lerner J. S. Small D. A. Loewenstein G. (2004). Heart strings and purse strings: carry-over effects of emotions on economic decisions. *Psychol. Sci.* 15 337–341. 10.1111/j.0956-7976.2004.00679.x 15102144

[B33] Loewenstein G. Lerner J. S. (2002). “The role of affect in decision making,” in *The Handbook of Affective Science*, eds Davidson R. J. Goldsmith H. H. Scherer K. R. (Oxford: Oxford University Press).

[B34] Loewenstein G. F. Weber E. U. Hsee C. K. Welch E. S. (2001). Risk as feelings. *Psychol. Bull.* 127 267–286. 10.1037/0033-2909.127.2.267 11316014

[B35] Manstead A. S. R. Wagner H. L. McDonald C. J. (1983). A contrast effect in judgments of own emotional state. *Motiv. Emot.* 7 279–290. 10.1007/BF00991678

[B36] Martin L. L. (2000). “Mood do not convey information: moods in context do,” in *Feeling and Thinking: The Role of Affect in Social Cognition*, ed. Forgas J. P. (Cambridge: Cambridge University Press), 153–177.

[B37] Murphy S. T. Monahan J. L. Zajonc R. B. (1995). Additivity of nonconscious affect: combined effects of priming and exposure. *J. Pers. Soc. Psychol.* 69 589–602. 10.1037/0022-3514.69.4.589 7473021

[B38] Neumann R. Seibt B. Strack F. (2001). The influence of mood on the intensity of emotional responses: disentangling feeling and knowing. *Cogn. Emot.* 15 725–747. 10.1080/02699930143000266

[B39] Neumann R. Strack F. (2000). “Experiential and non-experiential routes of motor influences on affect and evaluation,” in *The Message Within: Subjective Experiences and Social Cognition*, eds Bless H. Forgas J. P. (Philadelphia, PA: Psychology Press), 52–68.

[B40] Olsen G. D. Pracejus J. W. (2004). Integration of positive and negative affective stimuli. *J. Consum. Psychol.* 14 374–384. 10.1207/s15327663jcp1404_7

[B41] Ortony A. Clore G. L. Collins A. (1988). *The Cognitive Structure of Emotions.* New York, NY: Cambridge University Press.

[B42] Perrott D. A. Bodenhausen G. V. (2002). The way you make me feel: integral affective influences on interpersonal behavior. *Psychol. Inq.* 13 84–86.

[B43] Peters E. Burraston B. Mertz C. K. (2004). An emotion-based model of risk perception and stigma susceptibility: cognitive appraisals of emotion, affective reactivity, worldviews, and risk perceptions in the generation of technological stigma. *Risk Anal.* 24 1349–1367. 10.1111/j.0272-4332.2004.00531.x 15563300

[B44] Peters E. Dieckmann N. F. Västfjäll D. Mertz C. K. Slovic P. Hibbard J. (2009). Bringing meaning to numbers: the impact of evaluative categories on decisions. *J. Exp. Psychol. Appl.* 15 213–227. 10.1037/a0016978 19751072

[B45] Peters E. Slovic P. (2000). The springs of action: affective and analytical information processing in choice. *Pers. Soc. Psychol. Bull.* 26 1465–1475. 10.1177/01461672002612002

[B46] Peters E. Västfjäll D. Gärling T. Slovic P. (2006). Affect and decision making: a “hot” topic. *J. Behav. Decis. Mak.* 19 79–85. 10.1002/bdm.528

[B47] Pham M. T. (1998). Representativeness, relevance, and the use of feelings in decision making. *J. Consum. Res.* 25 144–159. 10.1086/209532

[B48] Pham M. T. (2007). Emotion and rationality: a critical review and interpretation of empirical evidence. *Rev. Gen. Psychol.* 11 155–178. 10.1037/1089-2680.11.2.155

[B49] Pham M. T. (2009). “The lexicon and grammar of affect-as-information in consumer decision making: the GAIM,” in *Social Psychology of Consumer Behavior*, ed. Wänke M. (Milton Park: Psychology Press), 167–200.

[B50] Pham M. T. Cohen J. B. Pracejus J. W. Hughes G. D. (2001). Affect monitoring and the primacy of feelings in judgment. *J. Consum. Res.* 28 167–188. 10.1086/322896

[B51] Rottenstreich Y. Hsee C. K. (2001). Money, kisses and electric shocks: on the affective psychology of probability weighting. *Psychol. Sci.* 12 185–190. 10.1111/1467-9280.00334 11437299

[B52] Russell J. A. (2003). Core affect and the psychological construction of emotion. *Psychol. Rev.* 110 145–172. 10.1037/0033-295X.110.1.145 12529060

[B53] Schwarz N. (2001). “Feelings as information: implications for affective influences on information processing,” in *Theories of Mood and Cognition: A User’s Guidebook*, eds Martin L. L. Clore G. L. (Hillsdale, NJ: Lawrence Erlbaum Associates), 159–176.

[B54] Schwarz N. (2012). “Feelings-as-information theory,” in *Handbook of Theories of Social Psychology*, eds Van Lange P. Kruglanski A. Higgins E. T. (Los Angeles, CA: Sage).

[B55] Schwarz N. Clore G. L. (1983). Mood, misattribution and judgments of well-being: informative and directive functions of affective states. *J. Pers. Soc. Psychol.* 45 513–523. 10.1037/0022-3514.45.3.513

[B56] Schwarz N. Clore G. L. (2004). Mood as information: 20 years later. *Psychol. Inq.* 14 296–303. 10.1080/1047840X.2003.9682896

[B57] Schwarz N. Clore G. L. (2007). “Feelings and phenomenal experiences,” in *Social Psychology: Handbook of Basic Principles*, 2nd Edn, eds Higgins E. T. Kruglanski A. (New York, NY: Guilford).

[B58] Schwarz N. Strack F. Kommer D. Wagner D. (1987). Soccer, rooms, and the quality of your life: mood effects on judgments of satisfaction with life in general and with specific life-domains. *Eur. J. Soc. Psychol.* 17 69–79. 10.1002/ejsp.2420170107

[B59] Seta J. J. Haire A. Seta C. E. (2008). Averaging and summation: positivity and choice as a function of the number and affective intensity of life events. *J. Exp. Soc. Psychol.* 44 173–186. 10.1016/j.jesp.2007.03.003

[B60] Siemer M. Reisenzein R. (1998). Effects of mood on evaluative judgments: influence of reduced processing capacity and mood salience. *Cogn. Emot.* 12 783–805. 10.1080/026999398379439

[B61] Slovic P. (2007). “If I look at the mass I will never act”: psychic numbing and genocide. *Judgm. Decis. Mak.* 2 79–95.

[B62] Slovic P. Finucane M. L. Peters E. MacGregor D. G. (2002). “The affect heuristic,” in *Heuristics and Biases: The Psychology of Intuitive Judgment*, eds Gilovich T. Griffin D. Kahneman D. (New York, NY: Cambridge University Press), 397–420.

[B63] Slovic P. Västfjäll D. (2010). Affect, moral intuition, and risk. *Psychol. Inq. Int. J. Adv. Psychol. Theory* 21 387–398. 10.1080/1047840X.2010.521119

[B64] Slovic S. Slovic P. (2015). The arithmetic of compassion. *The New York Times*, (p. SR10).

[B65] Storbeck J. Clore G. L. (2008). Affective arousal as information: how affective arousal influences judgments, learning, and memory. *Soc. Personal. Psychol. Compass.* 2 1824–1843. 25067943 10.1111/j.1751-9004.2008.00138.x PMC4110743

[B66] Strack F. Martin L. L. Stepper S. (1988). Inhibiting and facilitating conditions of the human smile: a nonobtrusive test of the facial feedback hypothesis. *J. Pers. Soc. Psychol.* 54 768–777. 10.1037/0022-3514.54.5.768 3379579

[B67] Strack F. (1992). “The different routes to social judgments: experiential versus informational strategies,” in *The Construction of Social Judgments*, eds Martin L. L. Tesser A. (Hillsdale, NJ: Lawrence Erlbaum Associates, Inc), 249–275.

[B68] Västfjäll D. Gärling T. Kleiner M. (2004). Preferences for mood, emotional reactions, and anticipated emotional reactions. *Scand. J. Psychol.* 45 27–36. 10.1111/j.1467-9450.2004.00375.x 15016276

[B69] Västfjäll D. Peters E. Slovic P. (2008). Incidental mood and charitable behavior. *Paper Presented at Society for Judgment and Decision Making meeting*, Chicago.

[B70] Västfjäll D. Slovic P. (2013). “Cognition and emotion in judgment and decision making,” in *Handbook of Cognition and Emotion*, eds Robinson M. D. Watkins E. R. Harmon-Jones E. (New York, NY: Guilford Press), 252–271.

[B71] Västfjäll D. Slovic P. Mayorga M. Peters E. (2014). Affect and charity is greatest for a single child: compassion fade in charitable giving. *PLoS ONE* 9:e100115 10.1371/journal.pone.0100115 PMC4062481 24940738

[B72] Västfjäll D. Slovic P. Mayorga M. (2015). Pseudoinefficacy: negative feelings from children who cannot be helped reduce warm glow for children who can be helped. *Front. Psychol. Decis. Neurosci.* 6:616 10.3389/fpsyg.2015.00616 PMC4434905 26042058

[B73] Verplanken B. Hofstee G. Janssen H. J. W. (1998). Accessibility of affective versus cognitive components of attitudes. *Eur. J. Soc. Psychol.* 28 23–36. 10.1002/(SICI)1099-0992(199801/02)28:1<23::AID-EJSP843>3.3.CO;2-Q

[B74] Vohs K. D. Baumeister R. F. Loewenstein G. (2007). *Do Emotions Help or Hurt Decision Making? A Hedgefoxian Perspective*. New York, NY: Russell Sage Foundation Press.

[B75] Weaver K. Garcia S. M. Schwarz N. (2012). The presenter’s paradox. *J. Consum. Res.* 39 445–460. 10.1086/66449

[B76] Wegener D. T. Petty R. E. Smith S. M. (1995). Positive mood can increase or decrease message scrutiny: the hedonic contingency view of mood and message processing. *J. Pers. Soc. Psychol.* 69 5–15. 10.1037/0022-3514.69.1.5 7643302

[B77] Winkielman P. Berridge K. C. (2004). Unconscious emotion. *Curr. Dir. Psychol. Sci.* 13 120–123. 10.1111/j.0963-7214.2004.00288.x

[B78] Wiss J. Andersson D. Slovic P. Västfjäll D. Tinghög G. (2015). The influence of identifiability and singularity in moral decision making. *Judgm. Decis. Mak.* 10 492–502.

[B79] Wyer R. S. Clore G. L. Isbell L. (1999). “Affect and information processing,” in *Advances in Experimental Social Psychology* Vol. 31 ed. Zanna M. (New York, NY: Academic Press), 1–77.

[B80] Yadav M. S. (1994). How buyers evaluate product bundles: a model of anchoring and adjustment. *J. Consum. Res.* 21 342–353. 10.1086/209402

[B81] Zajonc R. B. (1980). Feeling and thinking: preferences need no inferences. *Am. Psychol.* 35 151–175. 10.1037/0003-066X.35.2.151

[B82] Zajonc R. B. Murphy S. T. Inglehart M. (1989). Feeling and facial efference: implications of the vascular theory of emotion. *Psychol. Rev.* 96 395–416. 10.1037/0033-295X.96.3.395 2756066

